# Recurrent spontaneous pneumothorax in pregnancy – a case report and review of literature

**DOI:** 10.1080/20009666.2018.1472514

**Published:** 2018-06-12

**Authors:** Noman Lateef, Mustafa Dawood, Karn Sharma, Abubakar Tauseef, Muhammad Asadullah Munir, Erin Godbout

**Affiliations:** a Dow University of Health Sciences, Karachi, Pakistan; b Medicine, Greater Baltimore Medical Center, Towson, MD, USA; c Saba University School of Medicine, Saba, Dutch Caribbean; d Radiology Department, Dr. Ziauddin Hospital, Karachi, Pakistan

**Keywords:** Pneumothorax, chest tubes, video-assisted thoracoscopic surgery

## Abstract

Spontaneous pneumothorax in pregnancy is a rare and life-threatening condition. In this report, a case of spontaneous pneumothorax occurring at 34 weeks’ gestation in a healthy 34-year-old primigravida is described. She had typical complaints of chest pain and dyspnoea and diagnosis was made by chest X-ray which showed an extensive pneumothorax in the right side. Pneumothorax recurred twice over approximately three weeks. A caesarean section secondary to small pelvic parameters was scheduled with the chest tube *in situ* and a healthy 2.5 kg female infant was delivered. We discuss spontaneous pneumothorax during pregnancy and review the literature.

## 1. Introduction

Spontaneous pneumothorax is a potentially life-threatening pathology defined by the presence of air in the pleural cavity due to rupture of small apical blebs or bullae in the absence of a known significant pulmonary disease or trauma [1]. In pregnancy the prevalence of pneumothorax is low, with approximately only 56 cases reported in the literature previously [2]. Prompt and accurate diagnosis of pneumothorax is crucial as it can be affiliated with dyspnoea in pregnancy and lead to sudden respiratory comprise secondary to tension physiology [3].

## 2. Case presentation

A 34-year-old woman, G1P0, presented in the emergency department at 34 weeks of pregnancy. Her complaints were sudden onset right-sided pleuritic chest pain and shortness of breath. There was no history of trauma, nausea, fever, chills, or vomiting. She had no obstetric pathology and was a non-smoker. The past medical history was significant for right-sided pneumothorax in 2011. She had complained of right-sided chest pain and her symptoms resolved with supplemental oxygen therapy. Unfortunately, her medical or any imaging records were not available. Otherwise, her history was unremarkable for any other pulmonary disease.

**Figure 1.**
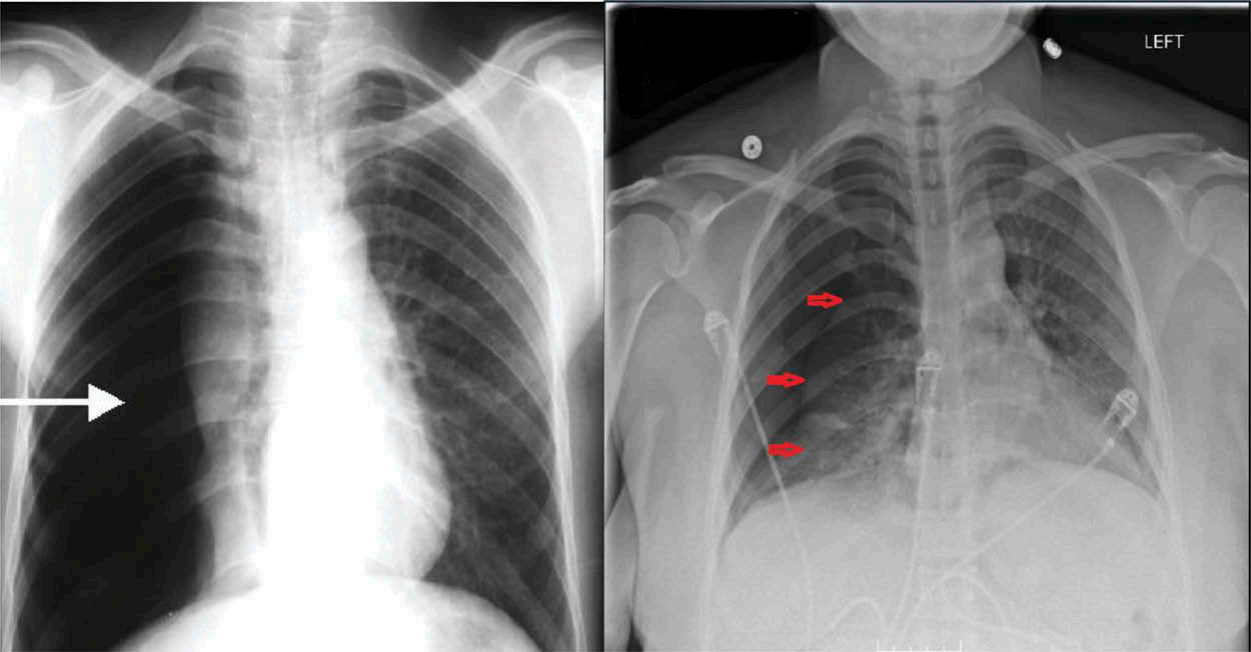
Chest X-ray showing large right pneumothorax with collapsed lung (*left*). Recurrence of right pneumothorax three weeks after the patient’s initial presentation (*right*).

Physical examination revealed a well-nourished woman with body mass index (BMI) of 36. Her blood pressure was 110/69 mm Hg, heart rate 105 bpm. She was found to be moderately tachypnoeic (respiratory rate 20 breaths per minute) with decreased air entry and had a hyperresonant percussion note over the right hemithorax. Peripheral pulse oximetry indicated normal oxygen saturation on room air. Blood gas analysis confirmed normal arterial oxygen and carbon dioxide tension. Chest radiograph revealed right-sided pneumothorax with partial collapse of the right lung (Figure 1).

A small-bore chest tube (pigtail catheter) was placed due to continued symptoms and was connected to low continuous suction. The patient was subsequently admitted to the medical floor as per cardiothoracic surgery recommendation. The patient gradually improved and the chest radiograph was repeated the next morning which showed resolution of pneumothorax. As the clinical condition had stabilised, the chest tube was removed and the patient was subsequently discharged.

**Figure 2.**
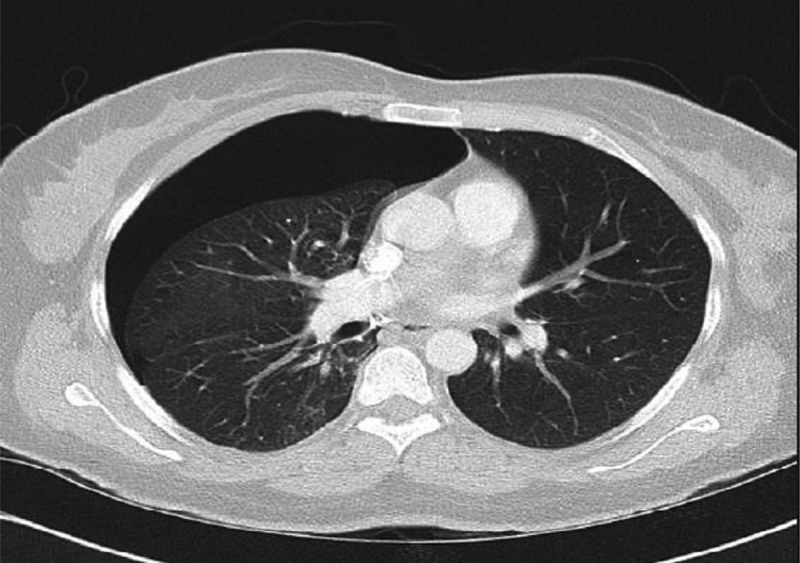
Chest CT revealed right-sided pneumothorax without any parenchymal abnormality.

The patient was readmitted three days later with another pneumothorax. Consequently, a computed tomography (CT) scan was performed which revealed right-sided pneumothorax without any parenchymal disease, confirming the diagnosis as primary spontaneous pneumothorax (Figure 2). A chest tube was reinserted and removed two days later following clinical improvement and resolution of the pneumothorax.

Unfortunately, the pneumothorax recurred after another two weeks and the chest tube was reinserted (Figure 1). But this time when the pneumothorax resolved, the multidisciplinary team decided to discharge the patient on atrium mini 500 ambulatory chest tube until the patients’ scheduled caesarean section secondary to small pelvis. Post discharge, the patient underwent her scheduled C-section and delivered a healthy female infant of weight 2.5 kg with good Apgar scores. No complications were seen afterwards.

The chest tube was removed after delivery, and video-assisted thoracoscopic surgery (VATS) was offered, but the patient declined the procedure. On a follow-up repeat chest X-ray no air leak or blebs were identified, hence, no active intervention was done. It was however decided that surgical intervention would be reconsidered should her pneumothorax recur.

## 3. Discussion

Primary spontaneous pneumothorax is described as air in the pleural space in otherwise healthy people without any lung disease. Cases reporting pneumothorax in pregnancy, particularly spontaneous pneumothorax are very few and rarely mentioned, as spontaneous pneumothorax rarely occurs in women compared with men. In 2007, a retrospective study of 250 spontaneous pneumothorax patients, identified only five cases as pneumothorax in pregnant patients [3]. Hence, due to low prevalence, the management guidelines regarding pneumothorax in pregnancy and vice versa, are still inadequate. Our patient first presented at 34 weeks of gestation. As noted from literature, the risk of recurrence of pneumothorax in pregnancy is high; with the majority occurring during the same pregnancy and during parturition [4,5]. Likewise, our patient had multiple recurrences during her perinatal period.

Rupture of a subpleural apical bulla or bleb is the most common cause of spontaneous pneumothorax in pregnancy, but pulmonary lymphangiomatosis (LAM) and cavitary tuberculosis (TB) have also been reported. Additional common risk factors include asthma, cocaine use, hyperemesis gravidarum, history of a previous pneumothorax, or an underlying infection [2,6], whereas, pulmonary ectopic deciduosis, ruptured hydatid cyst and alcohol use are also sparsely mentioned. During pregnancy and labour, the oxygen demand increases by 20 and 50%, respectively [7]. The functional residual capacity and total lung capacity are decreased in pregnancy, whereas respiratory rate, minute ventilation and tidal volume are increased, particularly contributed by raised progesterone level [3,8]. These changes in breathing pattern, in combination, are culpable for the higher risk of rupture of bullae and sub pleural bleb in pregnancy.

The typical symptoms of spontaneous pneumothorax, regardless of cause, include pleuritic chest pain associated with dyspnoea [2,4,9]. However, these typical symptoms are often attributed to paroxysmal tachycardia, neuralgia, or asthma exacerbation, thus contributing to underreporting of spontaneous pneumothorax [4]. Physical examination may show ipsilateral chest expansion, decreased tactile fremitus, tachycardia, tachypnea, cyanosis, diminished breath sounds, all on affected side.

Definitive diagnosis of pneumothorax can be established by chest radiograph, a standard two-view film confers exposure with 7 × 10^–5^ rad radiation, whereas the generally permitted cumulative dose of ionising radiation is <0.05 Gy = 5 rads [10]. Therefore, when one suspects pneumothorax in a pregnant patient, it is safe to proceed with the standard chest radiography with abdominal shield without placing the foetus at substantial risk from ionising radiation. If needed, shielded CT scan could also be performed as a useful imaging technique to clarify the underlying pathology and to help in operative planning when surgical treatment is indicated [7].

Review of 82 cases (including this case) showed that the patients were young (average age 26.6 years) and of low gravidity (mean gravidity 1.9). Pneumothorax occurred during the perinatal period in 54.8% and during the first or second trimester in 45.2% of cases. Initial treatment was observation only in 23.3%, tube thoracostomy in 73.1%, and thoracotomy in 3.6%. Of the entire aggregate of patients, 45.5% ultimately required thoracotomy for recurrence or persistence of the initial alveolar leak. The obstetric outcome was good, with 78.9% of patients having vaginal delivery, 19.9% having caesarean delivery, and one being foetal loss (1.2%). No neonatal complications were reported.

Multidisciplinary involvement including respiratory, obstetric, anaesthesiology and cardiothoracic surgery team is essential for the management of recurrent pneumothorax in pregnant women which is much similar to that in non-pregnant women. Initially, pneumothorax during pregnancy can be managed by simple observation if the mother is not dyspnoeic, there is no foetal distress and the pneumothorax is small (<2 cm). Otherwise aspiration can be performed; chest tube insertion being reserved for those with a persistent air leak. Patients with incomplete lung expansion after chest tube placement, bilateral pneumothoraces, recurrent pneumothorax or a hemopneumothorax require thoracotomy or VATS [11]. Optimal time for surgical management is during the second trimester. Chemical pleurodesis is also an option as a definitive management of recurrent pneumothorax with talc pleurodesis resulting in lowest recurrence rate [12]. Further, post-convalescence VATS procedure decreases the recurrence risk of pneumothorax in subsequent pregnancies, as successful pregnancies and spontaneous deliveries without pneumothorax recurrence have been reported in patients who underwent the procedure [11]. Our patient had her baby when she had the third chest tube in place, we believe that the delivery mitigated the pregnancy-related respiratory changes of the patient; thus, making the chest tube sufficient to treat the recurrence of the pneumothorax.

For concomitant obstetric management, it is recommended to avoid spontaneous delivery or caesarean section, as both have been associated with higher risk of increased intrathoracic pressure, which develops from the expulsive force generated by hyperventilation, coughing and valsalva manoeuvres during delivery and from positive pressure ventilation during caesarean section [7]. Also, nitrous oxide for intrapartum pain relief can compromise patient safety and is contraindicated, as it can cause and exacerbate a tension pneumothorax [13]. From previous data, the optimal mode of delivery will usually be that of elective assisted delivery (forceps or ventouse extraction) at or near term, with regional (epidural) anaesthesia [11]. Instrumental deliveries shorten the second stage of labour and thus reduce maternal expulsive efforts [8]. Similarly, if a caesarean section is unavoidable, then a spinal anaesthetic would be preferable to a general one, as it avoids the increased risk of pneumothorax resulting from positive-pressure ventilation, the use of nitrous oxide and adversities of extubation associated with general anaesthesia [3]. Based on the previous literature, there is no indication for caesarean section specifically related to spontaneous pneumothorax. The mode and time of delivery should only be based on obstetric parameters. So in this case we selected caesarean section as the mode of delivery because of patients’ small pelvis.

In terms of differential diagnosis for young women with pneumothorax, secondary causes such as pulmonary LAM and TB should be kept in mind. CT of the chest shows multiple round cysts at almost all fields of the bilateral lung for cases with LAM [6]. As for TB, radiological findings most commonly show cavitary lesions, however, among other lesions, consolidation (40%) and pulmonary infiltration (36%) were the most frequent [14]. As for diagnosis, biopsy of the cyst should be done if LAM is suspected and bacteriologic studies, including sputum smear for acid-fast bacilli can be done for TB [6,14]. Our patients’ CT did not reveal any parenchymal disease.

In conclusion, pneumothorax should be considered in pregnant patients particularly those with previous history and typical symptoms. Prompt radiographic confirmation is needed to prevent potential complications of maternal respiratory compromise, foetal hypoxia and preterm labour. Management of spontaneous pneumothorax in pregnancy is similar to that in general population. The best approach for delivery should be elective-assisted delivery at or near term. Caesarean section is not absolutely indicated and should be performed for obstetric reason only.
